# Prenatal Stress Inhibits Hippocampal Neurogenesis but Spares Olfactory Bulb Neurogenesis

**DOI:** 10.1371/journal.pone.0072972

**Published:** 2013-08-29

**Authors:** Laure Belnoue, Noelle Grosjean, Elodie Ladevèze, Djoher Nora Abrous, Muriel Koehl

**Affiliations:** 1 Inserm U862, Bordeaux, France; 2 Université de Bordeaux, Bordeaux, France; Duke University, United States of America

## Abstract

The dentate gyrus (DG) and the olfactory bulb (OB) are two regions of the adult brain in which new neurons are integrated daily in the existing networks. It is clearly established that these newborn neurons are implicated in specific functions sustained by these regions and that different factors can influence neurogenesis in both structures. Among these, life events, particularly occurring during early life, were shown to profoundly affect adult hippocampal neurogenesis and its associated functions like spatial learning, but data regarding their impact on adult bulbar neurogenesis are lacking. We hypothesized that prenatal stress could interfere with the development of the olfactory system, which takes place during the prenatal period, leading to alterations in adult bulbar neurogenesis and in olfactory capacities. To test this hypothesis we exposed pregnant C57Bl/6J mice to gestational restraint stress and evaluated behavioral and anatomic consequences in adult male offspring.

We report that prenatal stress has no impact on adult bulbar neurogenesis, and does not alter olfactory functions in adult male mice. However, it decreases cell proliferation and neurogenesis in the DG of the hippocampus, thus confirming previous reports on rats. Altogether our data support a selective and cross-species long-term impact of prenatal stress on neurogenesis.

## Introduction

The continuous generation of new neurons in the dentate gyrus (DG) of the hippocampal formation and in the olfactory bulb (OB) is hypothesized to maintain plasticity in these brain regions, supporting both structure and function [1], [2]. In the DG, neural precursors of the subgranular zone proliferate and give rise for the most part to glutamatergic granule neurons. In the OB, newborn neurons originate from neural progenitors located in the periventricular zone that migrate tangentially through the rostral migratory stream (RMS) to reach the OB where they differentiate largely in granular GABAergic neurons and, for a small part, in glomerular GABA and/or dopaminergic neurons [2], [3]. In both regions, almost half of the newborn cells die during the first steps of differentiation but the surviving ones are functionally integrated into the appropriate neuronal network [4], [5], [6], [7].

Many studies have addressed the functional role of these adult born neurons and it is now clearly established that neurogenesis in the hippocampus is involved in specific functions sustained by the DG [8], such as spatial learning and memory flexibility [9] or pattern separation [10], [11], [12], while bulbar newborn neurons were found to play a role in odor detection [13], perceptual learning [14], short- and long-term odor memory [13], [15], [16], or odor discrimination [14], [16], [17], [18].

Although evidence for continued neurogenesis are as numerous for the olfactory bulb than for the dentate gyrus, the range of factors that influence this process in the OB remains largely unknown compared to that described in the DG. In particular, the impact of early life events that occur during the critical phases of development, when both the OB and the DG get shaped, has been very sparsely studied in the OB but extensively detailed in the DG. Thus, both pre- and post-natal manipulations of the rearing environment were shown to disturb adult hippocampal neurogenesis [19]. In particular, prenatal stress (PS), a risk factor for the development of psychopathology [20] was found to decrease adult neurogenesis in the DG, reducing specifically cell proliferation rate without affecting neuronal or glial differentiation [21], [22], [23], [24], [25], [26]. As expected from the functional role of newborn hippocampal neurons, these modifications are paralleled by altered performances in spatial memory tests [22], [27], [28], [29], [30], [31].

Surprisingly, although the olfactory bulb and the stress system develop during the prenatal period, with the formation of the RMS and the delineation of the granular cells occurring at E18 [32], and the presence of glucocorticoid receptor mRNA detected as early as E17 in the OB [33], the impact of early life stress on adult bulbar neurogenesis and its associated functions remains unstudied. The first aim of this study was thus to test the hypothesis that prenatal stress could interfere with the normal course of SVZ-RMS-OB development, thereby affecting adult bulbar neurogenesis and its associated functions, i.e. olfactory learning and memory.

Furthermore, as all rodent studies showing an alteration of hippocampal neurogenesis and an impairment of cognitive performances have been performed on rat, the question of cross-species applicability of the effects of PS remains open. We thus tested whether prenatal stress decreases hippocampal cell proliferation in mice as it does in rats, an important question as this species provides genetic (transgenic and optogenetic) tools to test specific hypothesis related to mechanisms.

## Materials and Methods

### Animals

Three-month-old female C57Bl/6J mice (Charles River, France) were collectively housed under a 12 h light/12 h dark cycle (lights on from 8 am to 8 pm) in a temperature- (22+/−3°C) and humidity-controlled facility. Animals had *ad libitum* access to food and water. After 3 weeks of habituation to the housing conditions, females were mated with C57BL/6J males (Charles River, France) and pregnancy was determined by the presence of a vaginal plug checked once by day. Each female was removed from the breeding couple and individually housed upon plug detection (gestational day GD 0).

Experimental designs and procedures have been reviewed and approved by the local ethics committee (CEEA50 formerly known as “ethics committee on animal experimentation for Aquitaine and Poitou-Charentes region”). Studies were performed in Magendie Neurocenter (agreement B33096) in strict compliance with the institutional guidelines (council directive 87/848, October 19, 1987, Ministère de l'agriculture et de la Forêt, Service Vétérinaire de la Santé et de la Protection Animale) and international laws and policies (directive 86–609, 24 November 1986, European Community) effective at the time the experiments were carried out. Experimental work was performed under the responsibility of Dr. Muriel Koehl with agreement reference 3300061. All efforts were made to minimize suffering and reduce the number of animals used.

### Gestational stress

Gestational stress was carried out from day 12 of gestation until parturition (∼GD20). Females were restrained in plastic transparent cylinders (50 mL Falcon centrifuge tubes, 3 cm diameter, 11 cm long) for 45 min three times per day during the light cycle (9 am, 1 pm, 5 pm). Control mothers were left undisturbed throughout gestation. After delivery, dams and pups were undisturbed, except for cage change, until weaning (21^st^ day post-partum). At weaning, only males originating from litters containing at least 5 animals with an equal distribution between males and females were kept. Two groups were thus constituted: a Prenatally-Stressed group (PS) and a Not-Stressed group (NS). For each experimental group, a maximum of three pups per mother was used (Table 1) in order to sample an adequate number of litters and thus prevent any uncontrolled mother effect. Although studies in the biology of development field use the litter as the statistical unit, using the pup as the unit of analysis and sampling only a limited amount of pups per litter is a standard when regarding the long term consequences of the rearing environment [21], [24], [34], [35], [36], [37]. This procedure allows limiting the number of animals used for the experiment while granting a correct statistical sample, and is thus in accordance with the guidelines of the ethics committees.

**Table 1:** Number of animals studied and number of litters sampled for each experiment.

	*NS group*		*PS group*	
	*n*	*Nb of litters*	*n*	*Nb of litters*
Short-term memory test	8	4	8	6
Go/No Go task	13	9	12	9
CldU-IR cell nb in OB	28	23	22	21
CldU-IR cell nb in DG	28	21	21	20
DCX-IR cell nb in DG	29	21	21	20

### Thymidine analogue injections

**Figure 1:**
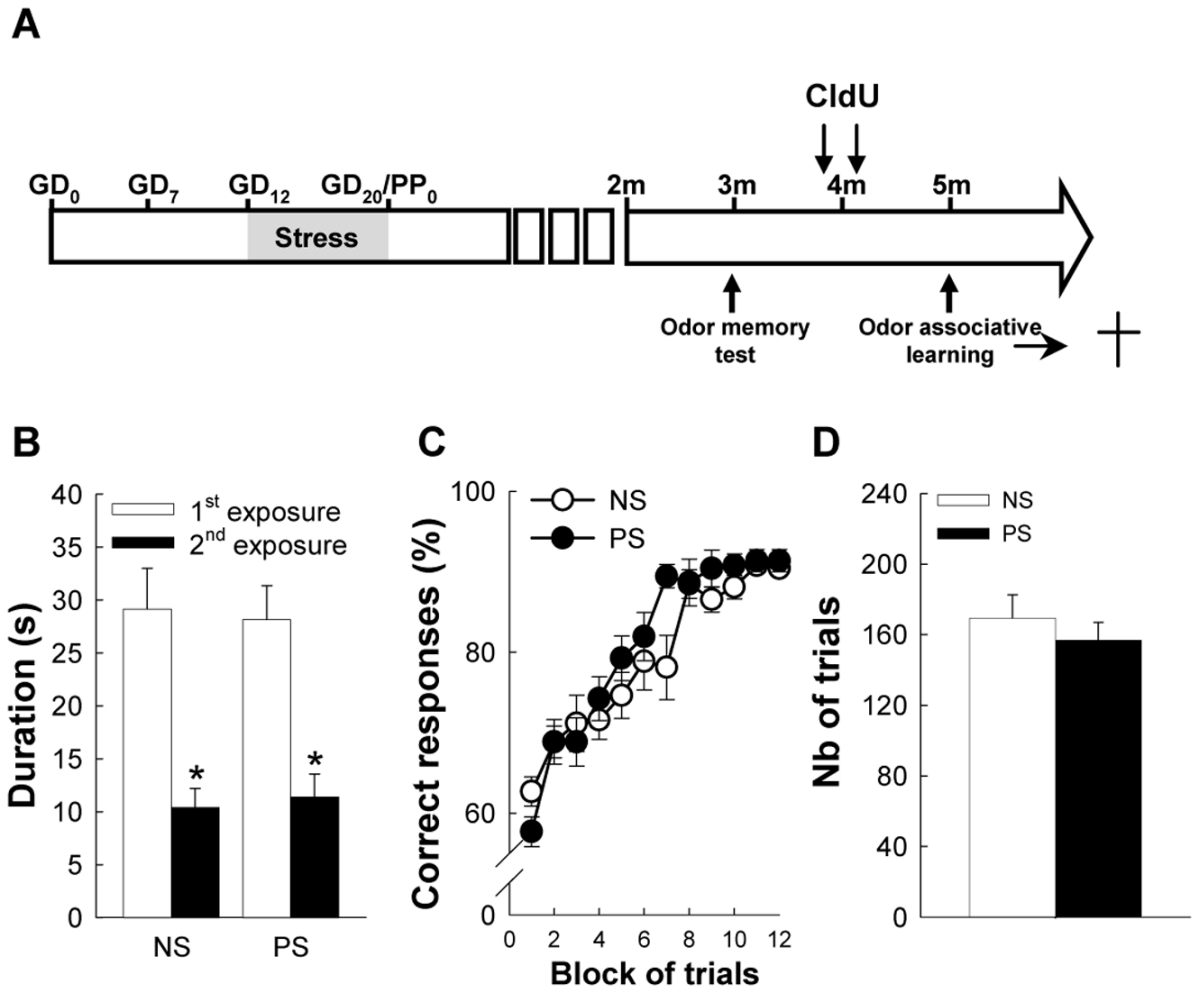
Prenatal stress has no impact on olfactory performances. (A) Scheme of the experiment. (B) Behavioral performances in a short-term memory task, n = 4 per group. (C) Behavioral performances in the olfactory discrimination learning task. (D) Number of trials to reach the criteria (2 consecutive blocks >85% successful trials) in the olfactory discrimination learning, n = 13 per group.

One group of NS and PS mice was injected with Chlorodeoxyuridine (CldU, Sigma), a marker of DNA synthesis (42.75 mg/kg, i.p., in 9 g/L saline solution) at a regimen of one daily injection on 2 consecutive days 5 weeks before starting the olfactory discrimination test (Figure 1A) in order to analyze long-term cell survival.

### Behavioral testing

An olfactory memory test was performed on 3-month old animals. One month later, the same mice were trained in an olfactory discrimination task based on associative learning, for which they were water-restricted (1,5 mL/day) 10 days before the beginning of the task. Each experimental group (NS and PS) was divided into three sub-groups: a control unmanipulated group (CC), a control water-restricted group (CCr), necessary to evaluate the impact of water-restriction on neurogenesis, and a paired group (P) that was water restricted and performed the discrimination task. No differences in immunohistochemistry results between the 3 sub-groups were detected (Table 2), so only results of P sub-groups are presented.

**Table 2:** Bulbar and hippocampal neurogenesis in control (CC), water restricted (CCr) and olfactory trained (P) control (NS) and prenatally-stressed (PS) mice.

	*NS group*			*PS group*			
	*CC*	*CCr*	*P*	*CC*	*CCr*	*P*	*ANOVA values*
Ki67-IR cell nb in SVZ	1205±114	1135±115	1194±70	1208±240	1073±123	1087±93	*Group effect, F_(1,41)_ = 0.32, p = 0.58*
							*Sub-group effect, F_(2,41)_ = 0.33, p = 0.72*
							*Group×Sub-group F_(2,41)_ = 0.11, p = 0.90*
CldU-IR cell nb in OB	25200±2755	18023±2219	22333±2080	18036±1996	22217±2053	19926±2994	*Group effect, F_(1,44)_ = 0.67, p = 0.42*
							*Sub-group effect, F_(2,44)_ = 0.15, p = 0.86*
							*Group×Sub-group F_(2,44)_ = 1.99, p = 0.15*
Ki67-IR cell nb in DG	1024±136	1050±154	1228±94	578±123	767±65	866±54	*Group effect, F_(1,46)_ = 15.19, p<0.001*
							*Sub-group effect, F_(2,46)_ = 2.53, p = 0.09*
							*Group×Sub-group F_(2,46)_ = 0.22, p = 0.80*
DCX-IR cell nb in DG	6704±335	7053±337	7083±253	5370±466	6231±230	5489±253	*Group effect, F_(1,44)_ = 22.28, p<0.0001*
							*Sub-group effect, F_(2,44)_ = 1.53, p = 0.23*
							*Group×Sub-group F_(2,44)_ = 0.86, p = 0.43*
CldU-IR cell nb in DG	3209±411	2029±323	2700±167	2200±406	2314±222	2040±121	*Group effect, F_(1,43)_ = 4.41, p<0.05*
							*Sub-group effect, F_(2,43)_ = 1.65, p = 0.20*
							*Group×Sub-group F_(2,43)_ = 2.89, p = 0.07*

#### Odor memory test

Testing took place in the mouse home cage. Odors were presented by placing a cotton swab impregnated with 5 µL of odorant solution through the rods of the mouse cage top, 10 cm above the cage floor. A test session consisted of two 5 minutes odor presentations of the same odor (Odor A: Decanal caprinaldehyde, 10^−3^M in mineral oil) with a 4 hours inter-trial interval. This interval was chosen based on pilot studies indicating that mice are able to recognize an odor presented 4 hours earlier (data not shown). Each mouse behavior during odor exposures was recorded and the time of cotton swab sniffing (snout 2 cm or less from the cotton swab) was scored off line by a trained experimenter blind of the mice group. A significant decrease in investigation time during the second presentation indicates that mice were able to recognize the odor previously presented.

#### Odor discrimination test

Using an olfactometer (Knosys, Lutz, USA), standard operant procedures [38] were used to train mice in a go/no-go discrimination task involving 2 very close odors: isoamylacetate and isoamylbutyrate (glomerular activity profiles can be found on http://gara.bio.uci.edu/index.jsp). Briefly, during the first 3 days, mice were trained to lick a water-reinforced tube (5 µl of water delivered per lick) when odor S^+^ (1% Isoamylacetate in mineral oil, 50 CC/min) was presented; during this training phase, animals underwent on average 90 trials per day; on the fourth day, i.e., the test day, the S^−^ stimulus (1% Isoamylbutyrate in mineral oil, 50 CC/min) was introduced, and animals learned to refrain from licking when S^−^ was presented. During this discrimination phase, trials S^+^ and S^−^ were presented in a random order, and testing was stopped when the animals performed two successive blocks (each block consists of 10 S^+^ and 10 S^−^ trials) of at least 85% of success. In order to study a difference in odor discrimination learning between PS and NS groups of mice, both the time course of correct responses over trial presentation and the total number of trials to reach the criteria have been evaluated for each group.

### Immunohistochemistry

Animals were sacrificed one hour after the odor discrimination test. They were anesthetized with pentobarbital (100 mg/kg) and perfused transcardially with 30 mL of PBS, pH = 7.3 and 30 mL of 4% paraformaldehyde in PB, pH = 7.3. Following a five-day postfixation period in paraformaldehyde, 40 µm sagittal sections were cut on a vibratome and collected in PBS-azide (0.02%). For CldU, Ki67 and DCX immunohistochemistries, sections were processed according to a standard procedure [39]. Briefly, for each staining, one in ten free-floating sections were incubated with a rat monoclonal anti-CldU (1/1000; Accurate), a rabbit monoclonal anti-Ki67 (1/2000, Novocastra) or a rabbit polyclonal anti-DCX antibody (1/4000, Sigma). Then, sections were incubated with biotin-labeled goat anti-rat (1/1000, Amersham) or goat anti-rabbit (1/400 and 1/500, for Ki67 and DCX, respectively, Dako) secondary antibodies. Immunoreactivities were visualized by the biotin-streptavidin technique (ABC kit; Dako) with 3,3′-diaminobenzidine as chromogen for CldU and Ki67 staining, and 3,3′-diaminobenzidine-Nickel chloride as chromogen for DCX.

For triple CldU-NeuN-S100 labeling, sections were treated with 2N HCl (30 min at 37°C) and incubated with a mixture of rat monoclonal anti-CldU antibody (1/500; Accurate), mouse monoclonal anti-NeuN antibody (1/1000, Millipore Bioscience Research Reagents) and rabbit polyclonal anti-S100 (1/2000, Sigma). Immunoreactivities were revealed with Cy3 goat anti-rat (1/1000, Jackson), Cy5 goat anti-mouse (1/1000, Molecular Probe), and Alexa 488 goat anti-rabbit (1/1000, Molecular Probe) secondary antibodies. Sections were mounted on glass slides and coverslipped with polyvinyl alcohol mounting medium with 1,4-diazabicyclo[2.2.2] octane (PVA-DABCO).

### Stereological analysis of staining

The number of positive cells was quantified in the left hemisphere under 1000× magnification with the optical fractionator method on a systematic random sampling of every tenth section along the temporo-median axis. For the OB, 50×50 µm counting frames at evenly spaced intervals of 300×300 µm, with an exclusion guard zone of 2 µm, was used to evaluate the number of CldU-IR cells (Stereo Investigator software, MicroBrightField, VT, USA). Ki67 immunoreactivity was quantified in the dorsolateral SVZ in two areas from each section; Stereoinvestigator software was used to measure the scored area and the density of Ki67 cells was calculated from the total number of Ki67 cells divided by the total scored area. For the DG, all CldU-, Ki67-, and DCX-IR cells were counted.

### Quantification of CldU-NeuN-S100-IR cells

All analyses were performed with a confocal microscope (Leica DMRTCS SP2 AOBS) equipped with a 63× oil-immersion objective, an argon laser (488 nm), a green helium-neon laser (543 nm) and a red helium-neon laser (633 nm). Confocal acquisitions were performed along the entire *z*-axis (25 µm) using 0.8 µm intervals and a 1× zoom for the DG, and a 1.6× zoom for the OB.

### Statistical analyses

All statistical analyses were performed with Statistica 8.0 software (Statsoft). For each experimental group, data were tested for normality using the Shapiro-Wilk test.

To detect a potential effect of water restriction and odor discrimination learning on neurogenesis, a 2-way ANOVA (group effect: NS, PS; sub-group effect: CC, CCr, and P) was performed. Odor memory and odor discrimination data were analyzed with two-way ANOVAs (group effect: NS, PS; time effect: 2 levels -1^st^ vs 2^nd^ exposure- for odor memory, 12 levels -block of trial- for odor discrimination). Immunohistochemistry data were analyzed with Student's *t* tests. All data are presented as mean ± SEM.

## Results

### Impact of prenatal stress on short term odor memory

We probed the olfactory capabilities of control and prenatally-stressed animals by first examining their short-term odor memory. To this end, the same odor (odor A) was presented twice to the animals with a 4 hours delay, and we found that odor exploration time was lower during the second exposure compared to the first one (Fig. 1B; exposure effect: *F*_(1,14)_ = 130.45, p<0.001) indicating that animals remember the odor previously presented. In addition, exploration time was similar in NS and PS mice (group effect: *F*_(1,14)_ = 0.0003, p = ns; group×exposure effect: *F*_(1,14)_ = 0.41; p = ns), thus indicating that prenatal stress did not disturb short-term odor memory.

### Impact of prenatal stress on odor discrimination

One month later, mice were tested in an olfactometer and their odor discrimination capabilities were evaluated using an associative learning paradigm and two closely related odors (Isoamylacetate and isoamylbutyrate, http://gara.bio.uci.edu). For 3 days, mice were trained to associate isoamylacetate with water reinforcement and on the fourth day, isoamylbutyrate was introduced, for which animals had to learn that it was not reinforced in a go/no-go procedure. Results showed that both groups readily improved their performances in the discrimination task (Fig. 1C; block effect, *F*_(11,264)_ = 49.24, p<0.0001). Although the time course analysis indicated an effect of stress (group×block *F*_(11,264)_ = 1.876, p<0.05), this effect was only due to a slightly reduced performance of NS mice during the 7^th^ block of trials (NK *post-hoc test*, p<0.05), and prenatal stress did not affect overall performances of the animals (group effect, *F*_(1,24)_ = 1.004, p = ns). The number of trials necessary to reach 85% of correct responses over 2 consecutive blocks confirmed this result, as NS and PS mice did not differ (Fig. 1D; t_24_ = 0.74, p = ns). Altogether these results showed that prenatal stress had no impact on olfactory discrimination tested in an associative learning paradigm.

### Effect of prenatal stress on bulbar adult neurogenesis

**Figure 2:**
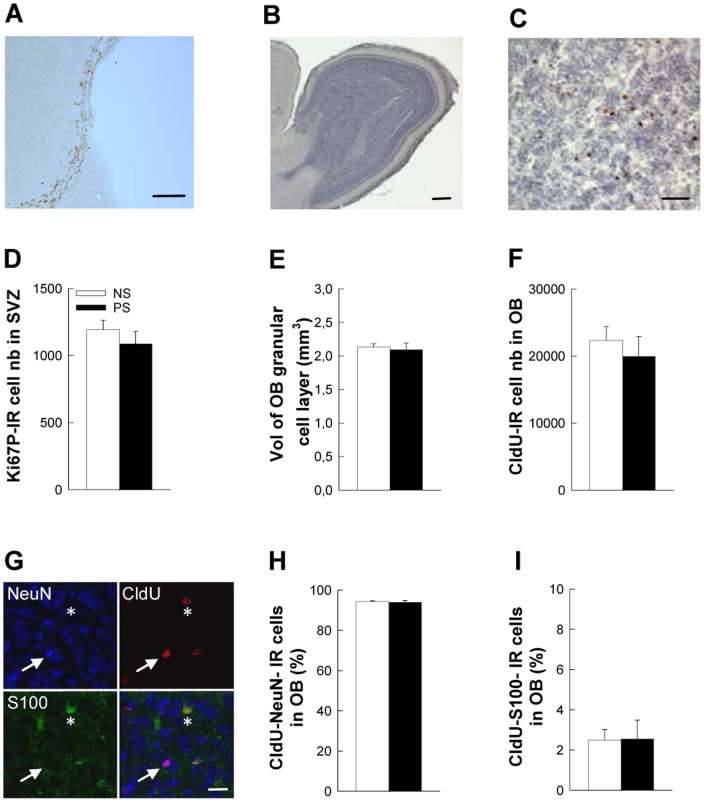
Prenatal stress does not disturb adult bulbar neurogenesis. (A) Illustration of Ki67-IR cells in the SVZ. (B) Sagittal view of the olfactory bulb. (C) High magnification of the granule cell layer of the olfactory bulb with 5-week-old CldU-IR cells. (D) Number of Ki67-IR cells in the dorsolateral corner of the SVZ, n = 9–12 per group. (E) Volume of the granule cell layer of the OB, n = 10–14 per group. (F) Number of 5-week-old CldU-IR cells in the GCL, n = 10–14 per group. (G) Illustration of CldU-NeuN-IR cells (white arrow) and of CldU-S100-IR cells (white star). (H) Percentage of CldU-NeuN-IR cells in the GCL, n = 5 per group. (I) Percentage of CldU-S100-IR cells in the GCL, n = 5 per group. Scale bars = 100 µm, 100 µm, 80 µm, and 20 µm for A, B, C, and G, respectively.

To first determine a potential effect of prenatal stress on the olfactory bulb, we evaluated cell proliferation in the dorso-lateral subventricular zone, where we could not evidence any effect of prenatal stress (Fig. 2A,D; t_19_ = 0.93, p = ns). We then determined whether long-term cell survival was affected by evaluating the number of 5-week old CldU-IR cells in the granule cell layer (GCL). As for cell proliferation, cell survival was unaffected by prenatal stress (Fig. 2C,F; t_19_ = 0.37, p = ns) and as a consequence the volume of the granule cell layer (GCL) was identical between groups (Fig. 2B,E t_22_ = 0.68, p = ns). Finally, we determined whether prenatal stress had an impact on neuronal or astroglial differentiation but no difference could be detected between NS and PS mice in the percentage of newborn cells endorsing a neuronal phenotype (CldU-NeuN-IR cells, Fig. 2G,H; t_8_ = 0.47, p = ns) or an astroglial phenotype (CldU-S100-IR cells; Fig. 2G,I; t_8_ = 0.07, p = ns).

### Impact of prenatal stress on hippocampal adult neurogenesis

**Figure 3:**
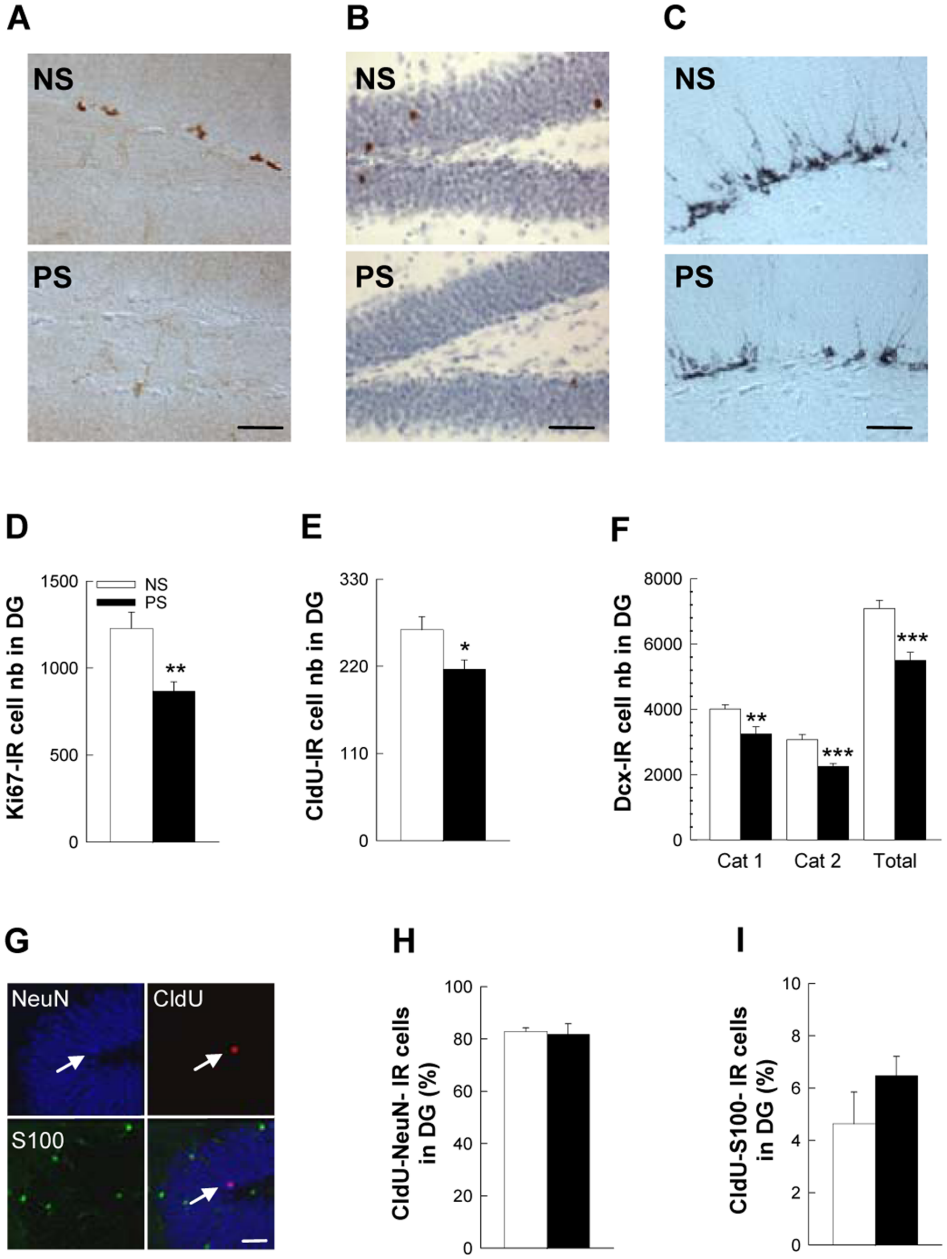
Prenatal stress decreases adult hippocampal neurogenesis. (A) Illustration of Ki67-IR cells in the DG in NS and PS groups. (B) Illustration of CldU-IR cells in the DG in NS and PS groups. (C) Illustration of DCX-IR cells in the DG in NS and PS groups. (D) Number of Ki67-IR cells in the DG, n = 10–14 per group. (E) Number of 5-week-old CldU-IR cells in the DG, n = 10–14 per group. (F) Number of DCX-IR cells in the DG, n = 10–14 per group. (G) Illustration of CldU-NeuN-IR cells (white arrow) and of CldU-S100-IR cells (white star). (H) Percentage of CldU-NeuN-IR cells in the DG, n = 5 per group. (I) Percentage of CldU-S100-IR cells in the DG, n = 5 per group. Scale bars = 100 µm, 100 µm, 50 µm, and 20 µm for A, B, C, and G, respectively.

It was previously shown by us and others that prenatal stress in rats decreases the number of adult-born neurons in the DG by limiting cell proliferation [22], [23], [26]. Similarly, we found here that cell proliferation, assessed by the number of Ki67-IR cells, was decreased in prenatally-stressed mice (Fig. 3A,D; t_22_ = 3.00, p<0.01).

The number of 5-week old surviving CldU-IR cells was also reduced in PS mice (Fig. 3B,E; t_22_ = 5.00, p<0.01), which could either reflect the decrease in cell proliferation and/or a decrease in cell survival rate. We thus calculated the rate of cell survival by dividing, for each animal, the number of 5-week-old CldU cells by the number of proliferating Ki67 cells. We found that this ratio was not modified by PS (data not shown, t_21_ = −0.397; p = ns), indicating that cell survival was not affected and that the decrease in the number of CldU-IR cells was du to the initial reduction in cell proliferation.

As expected from these results, the number of doublecortin-IR cells, a surrogate of neurogenesis, was also decreased in PS mice (Fig. 3C,F; t_22_ = 4.32, p<0.001). We subcategorized these DCX-IR cells according to their dendritic morphology, as it reflects different stages of neuronal maturation [40], [41]. Indeed, cells in an immature stage display a short or no process, and a horizontally-oriented nucleus, while morphologically mature cells display complex, tertiary dendrites protruding into the molecular layer, and a vertically-oriented nucleus. This analysis indicated that both categories are affected by prenatal stress (Fig. 3C; Cat1: t_22_ = 3.19, p<0.01, Cat 2: t_22_ = 4.11, p<0.01). Finally, we analyzed whether prenatal stress favored a specific maturation pathway of newborn cells and found that both neuronal (Fig. 3G,H; CldU-NeuN-IR cells) and astroglial (Fig. 3G,I; CldU-S100-IR cells) differentiation were unaffected by prenatal stress (t_8_ = 0.24 and −1.29, respectively for NeuN, and S100, respectively, both p = ns). Altogether these data show that prenatal stress reduces hippocampal neurogenesis by specifically targeting cell proliferation.

## Discussion

In this study, we showed that prenatal stress in mice has no impact on adult bulbar neurogenesis and olfaction measured through odor memory and odor discrimination, while it reduces hippocampal neurogenesis. These results indicate that although adult neurogenesis is a common feature of the olfactory bulb and the hippocampus, it is not controlled in both regions by the same environmental determinants.

### Prenatal stress does not affect odor memory and odor discrimination

In order to test the impact of prenatal stress on functions associated with adult bulbar neurogenesis, we have focused on short-term odor memory and discrimination of closely related odors in which newborn neurons have been implicated [13], [14], [16]. Contrary to our expectation, we found that prenatal stress had no long-term impact on these functions, at least in our experimental conditions. Deficits in olfaction have been reported for other prenatal insults, such as fetal alcohol exposure, which impairs the ability of adult mice to discriminate between similar odors [42], or prenatal cocaine exposure, which alters visual attention performances when olfactory distractors are presented [43], but to the best of our knowledge, our study is the first one to investigate olfaction in adult prenatally-stressed animals, so further studies will be needed to determine whether OB development shows a specific resistance to early life stress or whether more refine olfactory functions could be altered in prenatally-stressed animals.

### Prenatal stress does not affect olfactory bulb neurogenesis

In order to analyze the impact of prenatal stress on bulbar neurogenesis, we have assessed the level of cell proliferation in the subventricular zone, the number of surviving 5-weeks old cells in the olfactory bulb, and the rate of neuronal and glial differentiation of these cells. In accordance with the lack of effect of prenatal stress on olfactory functions, we found that none of these parameters was modified by prenatal stress. Although this suggests a resistance of the olfactory bulb to environmental challenges, other models of adult or perinatal interventions have highlighted the sensitivity of adult bulbar neurogenesis to environmental factors and life events. Thus in adulthood, it is now clearly established that modulating olfactory inputs (e.g. enriched environment, olfactory learning, or anosmia) modifies the level of bulbar neurogenesis [4], [6], [44], [45], [46], mainly by affecting newborn cell survival.

Data are much sparser regarding the impact of early life events. Thus if fetal alcohol exposure was found to decrease the number of dividing cells in the SVZ and of newly generated neurons in the OB, this effect was only transiently observed during the early stages of the development, and did not persist into adulthood [42]. Maternal separation was also found to decrease cell proliferation and increase cell death in the RMS immediately after or in the week following the separation phase [47]. Although this would indicate a possible reduction of cell proliferation in the SVZ and consequently a reduction of neurogenesis in the OB, these parameters have not been evaluated. Furthermore, adult brains were not analyzed, which does not allow concluding on the transientness or persistence of these effects. Finally, prenatal stress was found to decrease the number of neurospheres derived from neural stem cell in the developing and adult subependyma of the lateral ventricle, an effect that was confirmed by an *in vivo* reduction of cell proliferation in the SVZ in adult animals [48]. Although our results do not corroborate these findings, this study was performed on hamsters and we cannot exclude a potential species difference in the sensitivity of the SVZ-OB system to early life stress.

### Prenatal stress diminishes adult hippocampal neurogenesis

While we could not evidence any impact of prenatal stress on bulbar neurogenesis, we report that it decreases cell proliferation in the DG, leading to a reduction in the number of newborn neurons, and spares long-term cell survival and neuronal differentiation. To the best of our knowledge, our study is the first to investigate the impact of prenatal stress on adult neurogenesis in mice, and our results are consistent with the literature obtained in rats or primates [21], [22], [23], [36], [49] The extension of these findings to mice thus strengthens the relevance and robustness of this decrease.

### Differential effect of prenatal stress on bulbar and hippocampal adult neurogenesis

Altogether, our results show dissociation in the effects of early life events on bulbar and hippocampal neurogenesis. Such dissociation has already been reported. For example, voluntary running, which is one of the most potent activator of neurogenesis in the DG, has no impact on bulbar neurogenesis [50], confirming that neurogenesis in the OB and the DG are regulated by different external factors. Although the precise mechanisms involved in the deleterious effects of prenatal stress on hippocampal neurogenesis and the resistance of bulbar neurogenesis to the same life events are not elucidated, one likely candidate is the HPA axis, which is hyperactive in prenatally-stressed animals [21], [22], and more particularly glucocorticoids that may not have the same regulatory impact on the SVZ-OB system and the DG. Indeed, while it is now clearly established that corticosteroids reduce cell proliferation in the DG [51], [52], [53] the impact of stress and corticosteroids on bulbar neurogenesis is not as clear. Thus although a reduction of SVZ cell proliferation was reported after three weeks of chronic forced swim [54], or after corticosterone treatment at sub-physiological doses [55], chronic restraint and adrenalectomy were found inefficient on cell proliferation in the SVZ [56], [57].

In conclusion, the present study is the first one to indicate that prenatal stress applied during a critical period of development of the brain disrupts neurogenesis in the dentate gyrus of the hippocampus, but has no impact of bulbar neurogenic properties and their associated functions. Thus, although newborn neurons of both regions seem to be implicated in similar functions –involving different modalities- like learning and memory [13], [15], [16], [58], and pattern separation [59], they also seem to be set up and regulated by specific local cues. Uncovering the factors underlying the specific sensitivity of the DG, and resistance of the OB to prenatal stress should help us understand what defines and controls critical periods of brain development.
